# Trans-cerebral HCO_3_^−^ and PCO_2_ exchange during acute respiratory acidosis and exercise-induced metabolic acidosis in humans

**DOI:** 10.1177/0271678X211065924

**Published:** 2021-12-14

**Authors:** Hannah G Caldwell, Ryan L Hoiland, Kurt J Smith, Patrice Brassard, Anthony R Bain, Michael M Tymko, Connor A Howe, Jay MJR Carr, Benjamin S Stacey, Damian M Bailey, Audrey Drapeau, Mypinder S Sekhon, David B MacLeod, Philip N Ainslie

**Affiliations:** 1 Centre for Heart, Lung and Vascular Health, School of Health and Exercise Sciences, University of British Columbia Okanagan, Kelowna, BC, Canada; 2 Department of Anesthesiology, Pharmacology and Therapeutics, Vancouver General Hospital, University of British Columbia, Vancouver, BC, Canada; 3 Department of Cellular and Physiological Sciences, University of British Columbia, Vancouver, BC, Canada; 4 Department of Exercise Science, Physical and Health Education, Faculty of Education, University of Victoria, Victoria, British Columbia, Canada; 5 Department of Kinesiology, Faculty of Medicine, Université Laval, Québec, Canada; 6 Research Center of the Institut Universitaire de Cardiologie et de Pneumologie de Québec, QC, Canada; 7 Faculty of Human Kinetics, Department of Kinesiology, University of Windsor, Windsor, ON, Canada; 8 Neurovascular Health Laboratory, Faculty of Kinesiology, Sport and Recreation, University of Alberta, Edmonton, AB, Canada; 9 Neurovascular Research Laboratory, Faculty of Life Sciences and Education, University of South Wales, Pontypridd, UK; 10 Division of Critical Care Medicine, Department of Medicine, 8167 Vancouver General Hospital, Vancouver General Hospital, University of British Columbia, Vancouver, BC, Canada; 11 Human Pharmacology and Physiology Lab, Department of Anesthesiology, Duke University Medical Center, Durham, NC, USA

**Keywords:** Acidosis, bicarbonate, carbon dioxide, exercise, trans-cerebral exchange

## Abstract

This study investigated trans-cerebral internal jugular venous-arterial bicarbonate ([HCO_3_^−^]) and carbon dioxide tension (PCO_2_) exchange utilizing two separate interventions to induce acidosis: 1) acute respiratory acidosis via elevations in arterial PCO_2_ (PaCO_2_) (n = 39); and 2) metabolic acidosis via incremental cycling exercise to exhaustion (n = 24). During respiratory acidosis, arterial [HCO_3_^−^] *increased* by 0.15 ± 0.05 mmol ⋅ l^−1^ per mmHg elevation in PaCO_2_ across a wide physiological range (35 to 60 mmHg PaCO_2_; P < 0.001). The *narrowing* of the venous-arterial [HCO_3_^−^] and PCO_2_ differences with respiratory acidosis were both related to the hypercapnia-induced elevations in cerebral blood flow (CBF) (both P < 0.001; subset n = 27); thus, trans-cerebral [HCO_3_^−^] exchange (CBF × venous-arterial [HCO_3_^−^] difference) was reduced indicating a shift from net release toward net uptake of [HCO_3_^−^] (P = 0.004). Arterial [HCO_3_^−^] was *reduced* by −0.48 ± 0.15 mmol ⋅ l^−1^ per nmol ⋅ l^−1^ increase in arterial [H^+^] with exercise-induced acidosis (P < 0.001). There was no relationship between the venous-arterial [HCO_3_^−^] difference and arterial [H^+^] with exercise-induced acidosis or CBF; therefore, trans-cerebral [HCO_3_^−^] exchange was unaltered throughout exercise when indexed against arterial [H^+^] or pH (P = 0.933 and P = 0.896, respectively). These results indicate that increases and decreases in systemic [HCO_3_^−^] – during acute respiratory/exercise-induced metabolic acidosis, respectively – differentially affect cerebrovascular acid-base balance (via trans-cerebral [HCO_3_^−^] exchange).

## Introduction

The regulation of extracellular pH – which directly affects cells via local changes in intravascular or extravascular/interstitial conditions – is affected by rapid chemical buffering reactions, involving phosphate, glycolysis, and carbon dioxide tension (PCO_2_). The cerebral vasculature is exceptionally sensitive to changes in arterial PCO_2_ (PaCO_2_),^1–3^ such that cerebral blood flow (CBF) rapidly increases by 6–8% per mmHg increase in PaCO_2_ (reviewed in: Hoiland et al.^4^) This CBF responsiveness to PaCO_2_/[H^+^] acts to stabilize CO_2_ gradients and thus regulate pH across the blood-brain barrier (BBB).^4–6^ As CO_2_ travels rapidly across the BBB – indicated by the swift changes in CBF (<15–30 seconds) following stepwise changes in PaCO_2_^7–10^–perivascular/interstitial fluid (ISF) and intracellular brain tissue pH are tightly related to PaCO_2_. The Fick principle explains that elevated PaCO_2_ will be related to reductions in the trans-cerebral venous-arterial PCO_2_ difference as: 1) CBF varies directly with PaCO_2_,^1,3,11^ and 2) the cerebral metabolic production of CO_2_ ( $\dot{V}$ CO_2_) is unaltered in the physiological range; e.g., CBF = $\dot{V}$ CO_2_/PvCO_2_–PaCO_2_.^12,13^ Experimental results in several acid-base pathologies indicate the trans-cerebral venous-arterial PCO_2_ difference is reduced and increased with acute respiratory acidosis/alkalosis, respectively.^3,5,12,14–18^ These regulatory changes in the venous-arterial PCO_2_ difference contribute to tight regulation of a narrow range of cerebral interstitial pH^19,20^ and are influenced by the responsiveness of CBF to acute alterations in PaCO_2_; i.e., cerebrovascular CO_2_ reactivity.^6^

Exercise-induced metabolic acidosis results when the rate of ATP hydrolysis (H^+^ release) eventually exceeds the maximal buffering capacity; the excess CO_2_ is then removed via hyperventilation^21,22^ and these PaCO_2_ changes in part explain the CBF kinetics response with exercise.^23–25^ With exercise-induced acidosis, arterial [HCO_3_^−^] is markedly reduced due to excessive buffering of [H^+^]^21,22^ – however, whether this change in arterial [HCO_3_^−^] is related to alterations in trans-cerebral [HCO_3_^−^] and PCO_2_ exchange as well as *in vivo* buffering capacity has not been experimentally addressed in humans. The cerebral metabolic rate of oxygen (e.g., CMRO_2_ = CBF × arterial-venous oxygen content difference) increases linearly by up to 30% at maximal exercise intensities to support substrate utilization in the face of reductions in CBF.^26–33^ At maximal exercise, there is a higher relative contribution of cerebral anaerobic glycolysis and lactate oxidation.^34–36^ There is evidence to support that maximal exercise-induced acidosis facilitates trans-cerebral lactate exchange and related increases in lactate oxidation mediated via pH-sensitive transcellular [H^+^] gradients^37–39^ and increases in systemic lactate availability.^28,32,40–42^ Additionally, metabolic acidosis achieved with exhaustive exercise reportedly increases BBB permeability with direct relevance for transcellular CO_2_ transport.^38,43^ Overall, cerebrovascular acid-base regulation is likely interrelated with cerebral substrate prioritization during exercise via pH-sensitive utilization of lactate.^44,45^

The Henderson-Hasselbalch relationship (equation (2)) explains that acute elevations in arterial [HCO_3_^−^] would increase arterial buffering capacity; for example, with increases in arterial [HCO_3_^−^] any given change in PaCO_2_ will result in a lesser change in arterial [H^+^]/pH.^13,46^ This relationship would theoretically apply with reductions in arterial [HCO_3_^−^] and therefore be reflective of related decreases in arterial buffering capacity. Pre-clinical experiments in anesthetized and artificially ventilated cats show that rapid/transient [HCO_3_^−^]/Cl^−^ exchange occurs within 15 seconds between intravascular and extracellular fluid in response to elevated systemic arterial [HCO_3_^−^] at maintained PaCO_2_^47^ – these results indicate that extracellular [HCO_3_^−^] is partially and transiently altered with changes in arterial [HCO_3_^−^], however, such changes in extracellular [HCO_3_^−^] are restored (within 1 hour) and these [HCO_3_^−^]/Cl^-^ exchange kinetics are appreciably less influential than persistent and freely diffusible CO_2_ transport.^48,49^ At rest, cerebrospinal fluid (CSF) PCO_2_ – typically indexed via internal jugular venous sampling – is 6 to 11 mmHg higher and pH is 0.05 to 0.08 units lower than that of the arterial blood.^13,50,51^ As such, resting trans-cerebral net exchange of [HCO_3_^−^] and PCO_2_ (e.g., CBF × venous-arterial difference) is a positive value reflective of net release. A narrowing of the venous-arterial difference would indicate a shift toward net uptake and can be achieved, for example by: 1) larger increase in arterial relative to venous value; and 2) reduction in venous with a corresponding increase in arterial values. No study to date has investigated whether alterations in the trans-cerebral venous-arterial [HCO_3_^−^] and PCO_2_ differences during acute respiratory/exercise-induced metabolic acidosis are related to CBF and regulatory systemic changes in arterial [HCO_3_^−^] *in vivo* in humans.

The objective of this study was to investigate acid-base balance via alterations in trans-cerebral internal jugular venous-arterial [HCO_3_^−^] and PCO_2_ exchange utilizing two separate experimental interventions to induce acidosis: Study 1) acute respiratory acidosis via elevations in PaCO_2_ (range: 35 to 60 mmHg); and Study 2) metabolic acidosis via incremental cycling exercise to exhaustion (range: 7.45 to 7.20). We hypothesized that: 1) acute hypercapnic acidosis would *reduce* the venous-arterial [HCO_3_^−^] and PCO_2_ differences and this reduction would be related to higher CBF, less trans-cerebral [HCO_3_^−^] exchange, and elevated arterial [HCO_3_^−^]; and 2) arterial [HCO_3_^−^] would progressively decrease with maximal exercise-induced acidosis and – as explained by the non-linear CBF response with incremental exercise – this reduction in systemic [HCO_3_^−^] would be unrelated to any change in CBF or trans-cerebral venous-arterial [HCO_3_^−^] and PCO_2_ exchange.

## Methods

### Ethical approval

All participants provided informed written consent before participating in these studies. The original research studies were approved by the University of British Columbia Clinical Review Ethical Board (CREB: H16-01028, H18-01755, H15-00166, H11-03287) and the *Comité d’éthique de la recherche de l’Institut universitaire de cardiologie et de pneumologie de Québec* (CER: 21557) and according to the principles established by the Declaration of Helsinki (except for registration in a database). As the current study included a secondary analysis from previous institutional review board approved studies and the use of de-identified data sharing, this study did not require additional institutional review board review.

### Participants

These data were obtained during five separate experimental studies previously conducted at the University of British Columbia, Kelowna, British Columbia, Canada and the Université Laval, Québec City, Québec, Canada. Thirty-nine healthy adults completed the CO_2_ trials (n = 34 males and n = 5 females) and twenty-four healthy adults completed the exercise trials (n = 19 males and n = 5 females). Participants had no history of cerebrovascular, cardiovascular, or respiratory disease and were not taking any prescription medication at their time of participation. Participants refrained from alcohol and caffeine consumption as well as vigorous exercise or activity for at least 12 hours prior to testing.

### Experimental overview

The experimental questions addressed in this study involved *post-hoc* data analysis; therefore, few of the arterial and venous blood gas data have been reported separately in various context for previously published works from these studies; e.g.,.^36,52,53^ Importantly, the current experimental questions involved new data analyses that have not been previously reported.

### Study 1: Respiratory acidosis protocols

Participants completed one of the following protocols to elicit progressive stepwise steady-state elevations in PaCO_2_ via dynamic end-tidal forcing: 1) +3, +6, +9 mmHg PaCO_2_ (n = 11 males); 2) +4.5 and +9 mmHg PaCO_2_ (n = 12 males); 3) +8 mmHg PaCO_2_ (n = 5 females; n = 7 males); or 4) +10 and +20 mmHg PaCO_2_ (n = 4 males). The alterations in PaCO_2_ were calculated from the resting eupneic breathing end-tidal values. All measurements were taken after at least 2–3 minutes of steady-state. Previous investigations from our group have shown that cerebrovascular CO_2_ reactivity (utilizing the same dynamic end-tidal forcing system) is highly linear in the hypercapnic range up to +20 mmHg.^4,54^ Additionally, recent retrospective analysis by our research group^109^ has shown that following a rapid stepwise change in P_ET_CO_2_ (via the exact same experimental methods/techniques in the current studies), relevant cerebrovascular and cardiorespiratory variables achieve steady-state within 2-minutes of exposure duration to elevated inspired PCO_2_. As such, by applying a linear mixed model analysis, there is no statistical or physiological rationale to expect these results to be different whether participants completed the exact same stepwise steady-state elevations in PaCO_2_ compared to the varied stages utilized in this *post-hoc* analysis.

### Study 2: Exercise protocols

Participants completed supine incremental cycling exercise to exhaustion at various relative (0, 20, 40, 60, 80, 100% maximal workload; n = 12 males) and fixed exercise intensities (0, 50, 75, 100 watts; n = 5 females and 0, 75, 100, 125 watts; n = 7 males). Each workload was 3–5 minutes in duration and steady-state blood samples were drawn within the last 20 s of each exercise stage.

### Blood sampling

Approximately 1.0 ml of radial arterial and internal jugular venous blood were drawn at the same time into pre-heparinized syringes (SafePICO, Radiometer, Copenhagen, Denmark) and analyzed immediately using a commercial blood gas analyzer (ABL90 FLEX, Radiometer) at each experimental stage (n = 39). This analysis included measurements of PaCO_2_ and oxygen tension (PaO_2_), arterial oxygen saturation (SaO_2_), arterial oxygen content (CaO_2_), base excess, [H^+^], hemoglobin concentration ([Hb]), and pH. Data collected at the Université Laval were analyzed within 10−15 minutes for n = 12 (ABL800 FLEX, ABL825, Radiometer).

Arterial oxygen content (CaO_2_) was calculated as:

(1) $${\text{CaO}}_{\text{2}}(\text{mL}\cdot {\text{dL}}^{-\text{1}})=\left[\text{Hb}\right]\times \text{1}.\text{34}\times \left[{\text{SaO}}_{\text{2}}\left(\%\right)/\text{1}00\right]+0.00\text{3}\times {\text{PaO}}_{\text{2}}$$

Where 1.34 is the O_2_ binding capacity of hemoglobin and 0.003 is the solubility of O_2_ dissolved in blood.^55,56^

### Acid-base balance data analysis

Blood gas analyzers do not typically have the capacity to directly measure [HCO_3_^−^]; instead, it is calculated from measured PaCO_2_ and pH, by rearranging the Henderson-Hasselbalch equation (equations (2) and (3)).

(2) $$\text{pH}=\text{6.1}+\text{log}\;\frac{{\text{[HCO}}_{\text{3}}^{\text{-}}\text{]}}{\text{0.031}\times {\text{PCO}}_{\text{2}}}$$

(3) $${\text{[HCO}}_{\text{3}}^{\text{-}}\text{]}=\text{0.031}\times {\text{PCO}}_{\text{2}}\times {\text{10}}^{\text{(pH-6.1)}}$$

Where 6.1 is the pKa (i.e., −log of the acid dissociation constant) at 37.0°C^57^ and 0.031 mEq⋅l^−1^ per mmHg PCO_2_ is the solubility factor for dissolved CO_2_ plus carbonic acid (H_2_CO_3_) at 37.0°C in plasma.

The following calculations were used to quantify an index of *in vivo* buffering capacity termed “extracellular pH defense”.^58,59^ All *in vivo* buffering capacity calculations were analyzed with the average venous-arterial values of [HCO_3_^−^], [La], and pH as an estimate of cerebral tissue values. These results were consistent when expressed as arterial or internal jugular venous buffering capacity.

Exercise:

(4) $$\text{Δ}\text{[}{\text{HCO}}_{\text{3}}^{\text{-}}\;\text{]}\times \text{Δ}{\text{pH}}^{\text{-1}}$$

The reduction in [HCO_3_^−^] per unit pH is reportedly attributable to hyperventilation and HCO_3_^−^ buffering.^58^

(5) $$\text{-}\text{Δ}\text{[}\text{La}\text{]}\times {\text{pH}}^{\text{-1}}$$

This index includes lactate ([La]) and is considered total pH defense by hyperventilation, as well as HCO_3_^−^ and non-bicarbonate buffers.^58^

Respiratory acidosis:

(6) $$\text{-}\;\text{Δ}\text{[}{\text{HCO}}_{\text{3}}^{\text{-}}\;\text{]}\times \text{Δ}{\text{pH}}^{\text{-1}}$$

Equation (4) above has been adapted as [HCO_3_^−^] *increases* with respiratory acidosis.

### Cardiorespiratory

Detailed cardiorespiratory experimental methods and results have been previously reported elsewhere; e.g., literature.^36,52,53^ Briefly, the partial pressures of end-tidal CO_2_ and O_2_ (i.e., P_ET_CO_2_ and P_ET_O_2_, respectively) were controlled during acute respiratory acidosis using a custom-designed dynamic end-tidal forcing system to effectively regulate end-tidal gases across wide ranges of P_ET_CO_2_ and P_ET_O_2_,^60,61^ independent of ventilation ( $\dot{V}$ _E_).^62^ Beat-by-beat arterial blood pressure was continuously acquired via the radial artery pressure transducer positioned at the height of the right atrium (Edwards Lifesciences, TruWave VAMP, CA, USA) and the arterial blood pressure waveform was averaged to calculate MAP.

### Cerebrovascular

Detailed cerebrovascular experimental methods and results have been previously reported elsewhere; e.g., literature.^36,52,53^ Briefly, extra-cranial blood velocity and diameter of the internal carotid artery (ICA) and vertebral artery (VA) were measured using a 10-MHz multifrequency linear array Duplex ultrasound (Terason t3000; Teratech, Burlington, MA, USA). Pulse-wave mode was used to measure beat-to-beat peak blood velocity and arterial diameter was instantaneously measured via B-mode imaging; data were analyzed using custom edge-detection and wall tracking software (BloodFlow Analysis, version 5.1). The vessel location was decided on an individual basis to allow for reliable image acquisition, with the same location and consistent insonation angle (60°) repeated within participants and between trials.

Blood flow was calculated as:

(7) $$Q\;(\text{mL·min}-1)=\frac{\text{peak}\;\text{envelope}\;\text{blood}\;\text{velocity}}{2}\times (\pi (0.5\times \text{diameter}{)}^{2})\times 60$$

Total cerebral blood flow (CBF) was calculated as:

(8) $$\text{CBF}\;\text{(mL · }\text{min}-\text{1}\text{)}=\text{2}\times \text{(}Q_{\text{ICA}}+Q_{\text{VA}}\text{)}$$

Net [HCO_3_^−^] exchange was calculated as:

(9) $$\text{Net}\;{\text{[HCO}}_{\text{3}}^{\text{-}}\text{]}\;\text{exchange}\;\text{(mmol}\text{ × }{\text{min}}^{\text{−1}}\text{)}=\text{(}{\text{[HCO}}_{\text{3}}^{\text{-}}{\text{]}}_{\text{v}}\;\text{-}\;{\text{[HCO}}_{\text{3}}^{\text{-}}{\text{]}}_{\text{a}}\text{)}\times \text{CBF}$$

Net PCO_2_ exchange was calculated as:

(10) $$\text{Net}\;{\text{PCO}}_{\text{2}}\;\text{exchange}\;\text{(mmHg·}\text{min}\text{-}\text{1}\text{)}\;=\;\text{(}{\text{PvCO}}_{\text{2}}\text{-}{\text{PaCO}}_{\text{2}}\text{)}\times \text{CBF}$$

Where a negative value indicates a net uptake and a positive value indicates a net release.^63^

### Statistical analyses

All data are presented in-text as mean ± SD and as individual values in figures. Statistical analyses were performed using SPSS software (IBM statistics, Version 23.0) and Prism (GraphPad Software, Version 9.1.0). Normality was assessed using Shapiro-Wilk tests and by visual inspection of Q-Q plots. Statistical significance was set at P < 0.05. Relationships between select variables were analyzed using linear regression. A linear mixed-model analysis with fixed effects of either arterial [H^+^], pH, or PaCO_2_ was used to compare PCO_2_, [HCO_3_^−^], [La], *in vivo* buffering capacity, trans-cerebral exchange, and CBF separately for the respiratory acidosis and exercise interventions. Subjects were included as a random effect.

## Results

### Study 1: Respiratory acidosis

**Figure 1.**
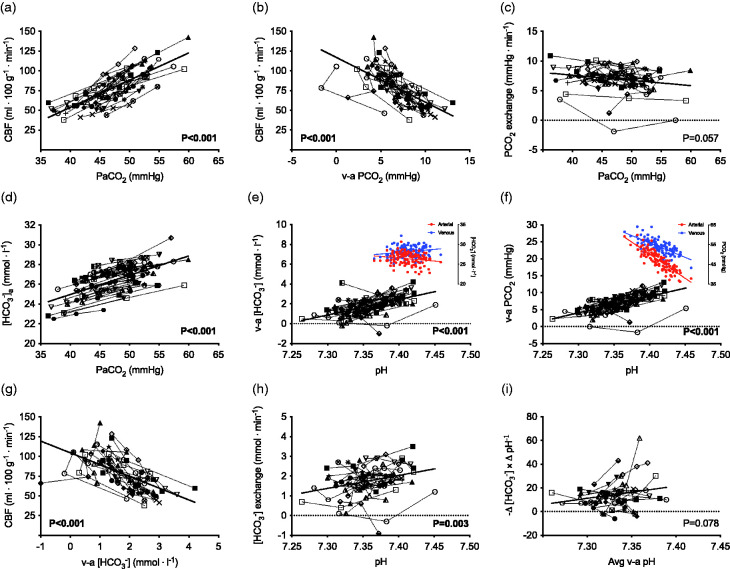
Venous-arterial [HCO_3_^−^] and PCO_2_ exchange with hypercapnic acidosis in humans. Total cerebral blood flow (CBF) increases with stepwise elevations in arterial PCO_2_ (PaCO_2_) (a); this hypercapnia-mediated increase in CBF is related to *narrowing* of the venous-arterial PCO_2_ difference (b) such that – explained via the Fick principle – trans-cerebral PCO_2_ exchange is unaltered with respiratory acidosis (c). Arterial [HCO_3_^−^] increases with progressive elevations in PaCO_2_ (d); this response may theoretically contribute to localized changes in CBF with severe respiratory acidosis – via increases in extravascular [HCO_3_^−^] – and thus, regulatory changes in [HCO_3_^−^] may help explain the maximal cerebrovascular vasodilatory reserve to PaCO_2_. There is a relationship between reductions in arterial pH (e.g., respiratory acidosis) and *narrowing* of the venous-arterial [HCO_3_^−^] (e) and PCO_2_ (f) differences. The reduction in venous-arterial [HCO_3_^−^] difference is achieved by a decrease in venous [HCO_3_^−^] and increase in arterial [HCO_3_^−^]; whereas, PaCO_2_ increases to a larger extent relative to PvCO_2_ with respiratory acidosis (illustrated by the coloured inlay figures). This narrowing is reflective of less [HCO_3_^−^] exchange (h) in part attributable to increases in [HCO_3_^−^] and CO_2_ ‘wash-out’ due to higher CBF with hypercapnia (g); i.e., the venous-arterial [HCO_3_^−^] and PCO_2_ differences were each related to increases in CBF (both P < 0.001; subset n=27). There was no relationship between the average venous-arterial *in vivo* buffering capacity (−Δ [HCO_3_^−^] × Δ pH^−1^) with stepwise respiratory acidosis when indexed against average venous-arterial pH (i). Data are individual values across stepwise progressive increases in PaCO_2_ for n = 27 (a, b, c, g, h, i) and n=39 (d, e, f) participants via dynamic end-tidal forcing.

Total CBF increased by 4 ± 1 ml ⋅ 100 g^−1^ ⋅ min^−1^ per mmHg elevation in PaCO_2_ across a wide range from 35 to 60 mmHg PaCO_2_ (P < 0.001; Figure 1(a)); this hypercapnia-mediated increase in CBF was related to *narrowing* of the venous-arterial PCO_2_ difference (P < 0.001; Figure 1(b)) such that trans-cerebral PCO_2_ exchange was unaltered with stepwise increases in PaCO_2_ (P = 0.057; Figure 1(c)). Arterial [HCO_3_^−^] increased by 0.15 ± 0.05 mmol ⋅ l^−1^ per mmHg elevation in PaCO_2_ (P < 0.001; Figure 1(d)). There was a relationship between respiratory acidosis and *narrowing* of the venous-arterial [HCO_3_^−^] and PCO_2_ differences; e.g., −0.16 ± 0.11 mmol ⋅ l^−1^ and −0.52 ± 0.22 mmHg reduction in venous-arterial [HCO_3_^−^] and PCO_2_ differences per nmol ⋅ l^−1^ increase in arterial [H^+^], respectively (both P < 0.001; Figure 1(e) and (f)). The venous-arterial [HCO_3_^−^] and PCO_2_ differences were each related to hypercapnia-induced increases in CBF (both P < 0.001; Figure 1(g)). As such, [HCO_3_^−^] exchange was reduced by -0.05 ± 0.08 mmol ⋅ min^−1^ per nmol ⋅ l^−1^ increase in arterial [H^+^]; i.e., less venous [HCO_3_^−^] efflux/release with respiratory acidosis (P = 0.004; Figure 1(h)).

### Study 2: Exercise-induced metabolic acidosis

**Figure 2.**
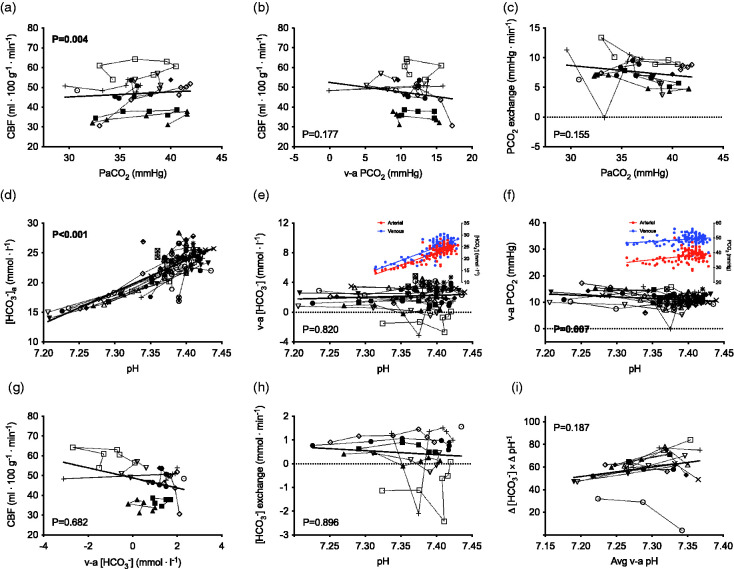
Venous-arterial [HCO_3_^−^] and PCO_2_ exchange with progressive submaximal to maximal cycling exercise. Total cerebral blood flow (CBF) is positively related to arterial PCO_2_ (PaCO_2_) throughout progressive cycling exercise to exhaustion (a); however, there is no relationship between CBF and the venous-arterial PCO_2_ difference (b), therefore, trans-cerebral PCO_2_ exchange is unrelated to PaCO_2_ during exercise (c). Arterial [HCO_3_^−^] is reduced with exercise-induced acidosis at maximal exercise (d). There is no relationship between the venous-arterial [HCO_3_^−^] difference and arterial pH (e) – as indicated by the equivalent reduction in arterial and venous [HCO_3_^−^] – however, there is *widening* of the venous-arterial PCO_2_ difference with exercise-induced acidosis (f) as indicated by a larger relative reduction in PaCO_2_ versus PvCO_2_ (illustrated by the coloured inlay figures). The exercise-induced CBF response was unrelated to the venous-arterial [HCO_3_^−^] difference (g); as such, there were no changes in trans-cerebral [HCO_3_^−^] *exchange* during exercise when indexed against arterial pH (h). There are no relationships between the average venous-arterial *in vivo* buffering capacity (Δ [HCO_3_^−^] × Δ pH^−1^ or −Δ [La] × Δ pH^−1^) during incremental cycling exercise to exhaustion (i). These data are reflective of the linear relationship between pH, reductions in [HCO_3_^−^], and increases in [La] with exercise-induced acidosis and indicate an unaltered *in vivo* buffering capacity at maximal cycling exercise. Data are individual values during supine incremental cycling exercise to exhaustion for n = 12 (a, b, c, g, h, i) and n=24 (d, e, f). Exercise stages included various relative (0, 20, 40, 60, 80, 100% maximal workload; n=12 males) and fixed exercise intensities (0, 50, 75, 100 watts; n = 5 females and 0, 75, 100, 125 watts; n=7 males). The *in vivo* buffering capacity was calculated at 60, 80, 100% maximal workload (I).

Total CBF was related to PaCO_2_ throughout progressive cycling exercise to exhaustion corresponding to 1 ± 1 ml ⋅ 100 g^−1^ ⋅ min^−1^ per mmHg change in PaCO_2_ (P = 0.004; Figure 2(a)). There was no relationship between CBF and the venous-arterial PCO_2_ difference (P = 0.177; Figure 2(b)); therefore, trans-cerebral PCO_2_ exchange was unrelated to PaCO_2_ during exercise (P = 0.155; Figure 2(c)). Arterial [HCO_3_^−^] was reduced by −0.48 ± 0.15 mmol ⋅ l^−1^ per nmol ⋅ l^−1^ increase in arterial [H^+^] with exercise-induced acidosis at maximal cycling exercise (P < 0.001; Figure 2(d)). There was no relationship between the venous-arterial [HCO_3_^−^] difference and arterial [H^+^] with exercise-induced acidosis (P = 0.801; Figure 2(e)). There was, however, a relationship between *widening* of the venous-arterial PCO_2_ difference by 0.12 ± 0.20 mmHg per nmol ⋅ l^−1^ increase in arterial [H^+^] during incremental cycling exercise to exhaustion (P = 0.006; Figure 2(f)); i.e., increased PCO_2_ uptake reflective of acidosis. The exercise-induced CBF response was unrelated to the venous-arterial [HCO_3_^−^] difference (P = 0.682; Figure 2(g)); as such, there were no changes in trans-cerebral [HCO_3_^−^] *exchange* during exercise when indexed against arterial [H^+^] or pH (P = 0.933 and P = 0.896, respectively; Figure 2(h)).

### In vivo buffering capacity during acute respiratory acidosis and exercise

Linear mixed-model analysis revealed no significant relationship between *in vivo* buffering capacity (−Δ [HCO_3_^−^] × Δ pH^−1^) and the average venous-arterial pH during stepwise respiratory acidosis (P = 0.078; Figure 1(i)). Additionally, there were no relationships between *in vivo* buffering capacity (Δ [HCO_3_^−^] × Δ pH^−1^ or -Δ [La] × Δ pH^−1^) and the average venous-arterial pH during incremental cycling exercise to exhaustion (P = 0.187 and P = 0.392, respectively; Figure 2(i)).

## Discussion

The results of this study indicate that trans-cerebral [HCO_3_^−^] and PCO_2_ exchange are differentially regulated in response to acute respiratory and exercise-induced acidosis. This finding is supported by: 1) acute hypercapnic acidosis elevated arterial [HCO_3_^−^]; however, trans-cerebral [HCO_3_^−^] exchange was *reduced* (i.e., indicating a shift from net release toward net uptake of [HCO_3_^−^]) and this was reflective in *narrower* venous-arterial [HCO_3_^−^] and PCO_2_ differences with higher CBF (Figure 1); and 2) arterial [HCO_3_^−^] was progressively reduced with maximal exercise-induced acidosis and this was unrelated to venous-arterial [HCO_3_^−^] and PCO_2_ exchange (Figure 2). Additionally, these results show there are no appreciable changes in the *in vivo* buffering capacity across a wide range of respiratory acidosis (up to +20 mmHg PaCO_2_) or incremental cycling exercise to exhaustion in humans (Figures 1(i) and 2(i)).

### Respiratory versus metabolic acidosis – influence of [HCO_3_^−^] exchange

The two experimental interventions of acute respiratory acidosis and exercise-induced metabolic acidosis each provoke equivalent reductions in pH with a markedly disparate arterial [HCO_3_^−^] response. For example, the reductions in arterial [HCO_3_^−^] during exercise are reflective of a 3-fold larger rate of change in [HCO_3_^−^] versus the small increases in [HCO_3_^−^] observed with respiratory acidosis (e.g., approx. -0.48 vs. +0.15 mmol ⋅ l^−1^ [HCO_3_^−^] per nmol ⋅ l^−1^ increase in [H^+^]). Additionally, it is noteworthy to consider how alterations in CMRO_2_ with exercise and respiratory acidosis will affect $\dot{V}$ CO_2_ and, therefore, PvCO_2_.^25,35,64–66^

Increases in PaCO_2_ facilitate rapid passive diffusion of CO_2_ across the BBB via reduced CSF driving pressure leading to increases in intra- and extracellular [H^+^], thus provoking acidosis through acid-base re-equilibration^67,68^ in accordance with Le Chatelier’s Principle (rightward shift in equation (11)).^69^ Overall, CO_2_ transport is the sum of the diffusion of 1) dissolved CO_2_; and 2) CO_2_ bound as HCO_3_^−^ (i.e., “facilitated CO_2_ diffusion”) – this process involves a flux of H^+^ equivalent to that of HCO_3_^−^ as per equation (11).^70–72^

(11) $${\text{CO}}_{\text{2}}+{\text{H}}_{\text{2}}\text{O}\text{CA}{\text{H}}_{\text{2}}{\text{CO}}_{\text{3}}↔{\text{HCO}}_{\text{3}}^{\text{-}}+{\text{H}}^{+}$$

Acute respiratory acidosis increases arterial [HCO_3_^−^] via continuous conversion of CO_2_ – rapidly catalyzed by carbonic anhydrase (CA) – shifting the equilibrium relationship toward the [HCO_3_^−^] buffer reaction. This is reflective of rapid increases in extracellular [HCO_3_^−^] due to intracellular [HCO_3_^−^] exchange with Cl^-^ to buffer the [H^+^] produced via CO_2_ hydration.^73^ Importantly, however, the ratio of arterial [HCO_3_^−^] to PaCO_2_ is lower such that pH is reduced (as per equation (2)).

At rest, the relative contributions of dissolved CO_2_, chemically bound to hemoglobin and protein carbamate, and HCO_3_^−^ to total CO_2_ exchange are approximately 5%, 10% and 85%, respectively. During severe exercise, HCO_3_^−^ facilitated CO_2_ diffusion is reduced to approximately 2/3 of total CO_2_ exchange (as the contribution of dissolved CO_2_ increases sevenfold with exercise-induced acidosis).^71,74^ With maximal exercise-induced acidosis, arterial [HCO_3_^−^] is markedly reduced due to compensatory buffering of [H^+^] leading to production of CO_2_ and H_2_O (leftward shift in equation (11)); the excess CO_2_ is then removed via hyperventilation.^21,22^

### Acute elevations in cerebral blood flow stabilize [HCO_3_^−^] and PCO_2_ gradients

Acute respiratory acidosis provokes three key regulatory responses: 1) arterial [HCO_3_^−^] increases (via inter-conversion of CO_2_) in response to elevated PaCO_2_ (Figure 1(d)); 2) there is rapid/transient exchange of HCO_3_^−^ and Cl^−^ across the BBB and between brain tissue and extracellular fluid;^47,73^ and 3) total CBF increases thus reducing the difference between arterial and extravascular PCO_2_, thereby limiting the rise in intracellular tissue PCO_2_ (Figure 1(b)). The increase in CBF in response to PaCO_2_ is restricted by the maximal vasodilatory response to hypercapnia *in vivo* in humans; e.g., +15–20 mmHg PaCO_2_ equates to 150% increase in CBF.^54^ Elevations in arterial [HCO_3_^−^] may theoretically contribute to localized changes in CBF with severe respiratory acidosis – via increases in extravascular [HCO_3_^−^] – and thus, regulatory changes in [HCO_3_^−^] may help explain the maximal cerebrovascular vasodilatory reserve to PaCO_2_. The internal jugular venous-arterial [HCO_3_^−^] difference is reduced with acute severe hypercapnic acidosis (Figure 1(e)) – a response likely explained by increased extravascular HCO_3_^−^ ‘wash-out’ as the reduction in trans-cerebral [HCO_3_^−^] difference is related to the hypercapnic CBF response (P < 0.001; Figure 1(g)). Arvidsson and colleagues (1981) showed that intravenous infusion of NaHCO_3_ in hypercapnic dogs causes a 50–70% reduction from the PaCO_2_-induced higher CBF. Additionally, cerebrospinal fluid [HCO_3_^−^] ([HCO_3_^−^]_CSF_) was appreciably higher following NaHCO_3_; these data indicate that acutely elevated PaCO_2_ may facilitate the transport of exogenous HCO_3_^−^ across the BBB (via higher CBF) resulting in cerebral vasoconstriction due to increased extravascular pH.^75^ The present results support that the CBF response to acute respiratory acidosis contributes to tight regulation of cerebrovascular pH (via narrowing of the trans-cerebral [HCO_3_^−^] and PCO_2_ differences) *in vivo* in humans.

### Regulation of cerebrovascular acid-base balance during exercise

Trans-cerebral venous-arterial [HCO_3_^−^] exchange was unaffected by maximal exercise-induced acidosis (e.g., pH 7.20; Figure 2(e)) and there was a *widening* of the venous-arterial PCO_2_ difference during incremental cycling exercise to exhaustion (Figure 2(f)). Total CBF increases steadily by 10−20% up to intensities of approximately 60–70% of maximal oxygen uptake ( $\dot{V}$ O_2max_) to regulate cerebral substrate delivery,^32,76^ and is mediated via relative alveolar hypoventilation (i.e., elevations in PaCO_2_) and increases in CMRO_2_.^25,36,77^ With progressive increases in cycling exercise intensity, the relatively linear CMRO_2_ response is coupled to cerebral oxygen delivery (CBF × CaO_2_) and oxygen extraction (CaO_2_ – CvO_2_/CaO_2_) × 100%)) rather than CBF *per se*. At maximal exercise, hyperventilatory-induced reductions in CBF – together with marked acidosis – would conceivably adversely affect intracellular/extravascular [HCO_3_^−^] and CO_2_ ‘wash-out’. Previous work by Bisgard and colleagues (1978) reported unaltered [HCO_3_^−^]_CSF_ and PaCO_2_-mediated *increases* in CSF pH (via hyperventilatory response) with severe near-maximal exercise in ponies^78^ – these data emphasize the importance of the ventilatory response on cerebrovascular PCO_2_/[HCO_3_^−^] regulation during exercise.^6^ Additionally, albeit during resting conditions, several studies report that sustained changes in CSF/extracellular [HCO_3_^−^] respond slowly (e.g., several hours) and only partially to systemic changes in arterial [HCO_3_^−^],^50,79–84^ thus explaining the steady [HCO_3_^−^]_CSF_ during acute exercise (<10 minutes). Taken together with the remarkably consistent trans-cerebral [HCO_3_^−^] exchange (Figure 2(h)) and *in vivo* [HCO_3_^−^] and [La] buffering capacity throughout exercise (Figure 2(i)), these data indicate that the CBF response to exercise *per se* likely plays a lesser role in cerebrovascular acid-base regulation (versus respiratory acidosis, for example).

### Experimental considerations

A key strength of this study was the invasive direct Kety-Schmidt technique to quantify trans-cerebral venous-arterial exchange of [HCO_3_^−^], PCO_2_, and [H^+^] *in vivo* in healthy humans; we recognize that this relies on the assumption that these sampling sites are reflective of *trans-cerebral exchange* due to practical and ethical experimental constraints.^85^ Additionally, the Duplex ultrasound derived indexes of extra-cranial blood flow utilized in the current experiment are assumed to indicate cerebral tissue nutritive flow. Although we verified PaCO_2_ values with blood gas sampling, without temperature correcting to adjust for higher blood temperature during exercise, we may have underestimated PaCO_2_ by 1–2 mmHg (due to reduced CO_2_ solubility at higher temperatures).^86,87^ Additionally, the influences of exercise^88–90^ and temperature^91–95^ may conceivably have a small effect on PCO_2_, H^+^, and HCO_3_^−^ via alterations in carbonic anhydrase activity in the exercise trial^96,97^ – however, this is unlikely to differentially affect the trans-cerebral venous-arterial exchange values *within* participants during this trial.

A subset of female participants completed the +8 mmHg PaCO_2_ respiratory acidosis protocol as well as the fixed intensity submaximal exercise protocol (n = 5 females and n = 7 males). Previous literature is equivocal, with studies reporting that cerebrovascular CO_2_ reactivity is higher in females,^98–100^ higher in males,^101–103^ or not different between sexes.^104,105^ Whether sex-related differences in cerebrovascular CO_2_ reactivity exist between females and males in response to a fixed targeted elevation in PaCO_2_, this is unlikely to affect our results as we saw no change in the *in vivo* buffering capacity (Δ [HCO_3_^−^] × Δ pH^−1^) when indexed against the average venous-arterial pH during respiratory acidosis. These results indicate that any within-subject change in the sensitivity of CBF to PaCO_2_ would be reflected in an equivalent pH response (with which we indexed against our dependent variables). Additionally, recent studies have shown no difference in the CBF response to moderate-intensity exercise between females and males^106,107^ with sex-related differences in CBF regulation only apparent at severe intensity exercise (80–100% maximal workload).^108^ As such, we do not expect that the inclusion of 5 female participants in the fixed intensity submaximal exercise protocol (0, 50, 75, 100 watts) would affect the variability of our results. Future investigations are merited to address any sex-related differences in cerebrovascular acid-base regulation in response to respiratory and exercise-induced metabolic acidosis.

## Conclusion

Taken together, these results indicate that increases and decreases in systemic arterial [HCO_3_^−^] – during acute respiratory/exercise-induced metabolic acidosis, respectively – differentially contribute to cerebrovascular acid-base balance. The previously recognized tight regulation of cerebral interstitial pH during acute hypercapnic acidosis^19,20^ is likely attributable to: 1) narrower internal jugular venous-arterial [HCO_3_^−^] and PCO_2_ differences; 2) higher CBF; and 3) reductions in trans-cerebral [HCO_3_^−^] exchange indicating less venous [HCO_3_^−^] efflux/release. These results are supportive of unaltered trans-cerebral [HCO_3_^−^] exchange with exercise-induced acidosis – consistent with the unchanged *in vivo* buffering capacity – which was unrelated to the CBF response throughout progressive cycling exercise to exhaustion.
